# CRISPR/Cas Genome Editing in Potato: Current Status and Future Perspectives

**DOI:** 10.3389/fgene.2022.827808

**Published:** 2022-02-02

**Authors:** Jagesh Kumar Tiwari, Tanuja Buckseth, Clarissa Challam, Rasna Zinta, Nisha Bhatia, Dalamu Dalamu, Sharmistha Naik, Anuj K. Poonia, Rajesh K. Singh, Satish K. Luthra, Vinod Kumar, Manoj Kumar

**Affiliations:** ^1^ ICAR-Central Potato Research Institute, Shimla, India; ^2^ ICAR-Central Potato Research Institute, Shillong, India; ^3^ School of Bioengineering and Biosciences, Lovely Professional University, Phagwara, India; ^4^ School of Biotechnology, Shoolini University, Solan, India; ^5^ ICAR-National Research Centre for Grapes, Pune, India; ^6^ ICAR-Central Potato Research Institute, Meerut, India

**Keywords:** abiotic, biotic, CRiSPR/Cas, genome editing, Potato, quality

## Introduction

Potato (*Solanum tuberosum* L.) (2*n* = 4*x* = 48) is the third most important food crop after rice and wheat in terms of human consumption. Potato is considered as the staple food in Europe and parts of Americas. In 2018, the world total potato production was 368.17 million tonnes led by China (90.26 mt) followed by India (48.53 mt) (FAOSTAT, 2018). The increasing world population from the now 7.7 to the expected 9.7 billion by 2050 has posed a great challenge of food availability (United Nations, 2019). Potato suffers from various pathogens, insect pests, and environmental abiotic stresses. The condition is worsening under the climate change scenario. In India, the mean potato productivity in major potato-growing states, which together account for about 90% of the national potato production, is likely to decline by 2.0% in 2050s and 6.4% in 2080s (Rana et al., 2020). To address these issues, conventional breeding has shown key roles in varietal development programs combined with the deployment of marker-assisted selection mainly for late blight, viruses, and potato cyst nematode–resistant varieties the world over such as Kufri Karan in India (ICAR-CPRI Annual Report, 2018-19). Later, potato transgenics have also been developed for resistance to diseases (e.g., late blight and viruses), abiotic stresses (e.g., heat and drought), insect pest (e.g., potato cyst nematode and potato tuber moth), processing quality (e.g., reduced cold-induced sweetening), but none of them are being applied at the field level. Hence, with the advancement of sequencing technologies and availability of the potato genome sequence (Potato Genome Sequencing Consortium, 2011), it is possible to modulate the target genes applying genomics tools like genome editing.

Genome editing is an advanced genomics tool which can be deployed for crop improvement by gene knock-out and insertion/deletion mutagenesis (Hameed et al., 2018). It allows double-stranded breaks (DSBs) at specific sites in the genome and repairs via naturally occurring DNA repair mechanisms, namely, nonhomologous end joining (NHEJ) or homologous recombination (HR). In the past, this system was earlier facilitated by protein-guided nucleases such as zinc finger nucleases (ZFNs) and transcription activator-like effector nucleases (TALENs). But now, attention has been driven on the new RNA-guided nuclease called clustered regularly interspaced short palindromic repeats (CRISPR)—CRISPR associated (Cas) (Nadakuduti et al., 2018). The TALENs and ZFNs require particular expertise, longer timelines, and higher costs than those needed for assembling CRISPR/Cas. Indeed, a tremendous progress has been reported on the utility of CRISPR/Cas in crops. In potato, CRISPR/Cas has been demonstrated for tuber quality, disease resistance (late blight and potato virus Y), phenotype, and other traits (Dangol et al., 2019; Hameed et al., 2020; Hofvander et al., 2021). This article provides the current status of CRISPR/Cas, future perspectives, and challenges in potato.

## CRISPR/Cas Genome Editing and Its Need in Potato

CRISPR/Cas is the most powerful biological tool to create targeted modification in the genome, which allows easy designing and construction of gene-specific single guide RNA (sgRNA). The sgRNA vectors are easily reprogrammable to direct *Streptococcus pyogenes* Cas9 (SpCas9) to generate DSBs and are then repaired endogenously by the error-prone NHEJ or HR pathways. CRISPR/Cas is divided into two distinct classes based on the sequence, structure, and functions of the Cas proteins. Class 1 consists of types I, III, and IV andutilizes a multi-protein effector complex, whereas class 2 includes types II, V, and VI and uses a single effector protein; of which, type II and V target DNA, whereas type VI targets RNA. CRISPR/Cas9 (class 2, type-II) is the most commonly exploited machinery for DNA target. Remarkable innovations in CRISPR/Cas9 variant FnCas9 (*Francisella novicida*) (Price et al., 2015) and CRISPR/Cas13a (type VI, LshCas13a from *Leptotrichia shahii*) (Aman et al., 2018) have opened new avenues for RNA targets also. The SpCas9 and RNase III ribonucleases generate the Cas9/guide RNA complex that recognizes and cleaves DNA sequences adjacent to the 5′-NGG protospacer adjacent motif (PAM) and induces site-specific DSBs (Khatodia et al., 2016; Cao et al., 2020). Currently, CRISPAR/Cas9 has revolutionized plant research due to its simplicity, multiplexing, cost-effectiveness, high efficiency, and minimum off targets. Unlike genetically modified organisms, CRISPR/Cas creates alterations in the existing genome without the introduction of foreign genes, particularly site-directed nucleases (SDN1 and SDN2). Hence, CRISPR/Cas is expected to be transgene free, and biosafety regulations are under consideration in various countries (Schmidt et al., 2020).

Several complex traits of agronomic importance are considered in potato while breeding a new variety. The multigenic-controlled biotic/abiotic stresses are difficult to improve through conventional breeding in less time, which could be possible by using CRISPR/Cas9. The gene knockout mechanism has been applied in potato for late blight resistance using susceptibility (S) genes (*StDND1*, *StCHL1*, and *StDMR6-*1) (Kieu et al., 2021). A few successful examples are discussed later for biotic/abiotic stress resistance/tolerance, tuber quality, and phenotype traits improvement in potato (Table 1, and Supplementary Figures 1 and 2).

**TABLE 1:** Successful examples of application of CRISPR/Cas genome editing technology for biotic and abiotic stress resistance/tolerance, tuber quality, and phenotype and other traits in potato.

Target gene	Trait	CRISPR system	Delivery/transformation system	Genotype	Key findings	Reference
Biotic stress resistance						
*P3*, *CI*, *NIb*, or *CP* (RNA virus genes)	PVY, PVS, and PVA resistance	LshCas13a	*Agrobacterium*	Desiree	Multiple PVY strain–resistant mutants	Zhan et al. (2019)
*StDND1*, *StCHL1* and *StDMR6-*1 (S-genes: Susceptibility genes)	Late blight resistance	Cas9	*Agrobacterium*	Desiree	Tetra-allelic mutants by knockout of *StDMR6-1* and *StCHL1* genes	Kieu et al. (2021)
*Caffeoyl-CoA O-methyltransferase* (*StCCoAOMT*)	Late blight resistance	Cas9	*Agrobacterium*	Russet Burbank	Increase in late blight resistance than control	Hegde et al. (2021)
Abiotic stress tolerance						
*StMYB44* (MYB transcription factor)	Phosphate transport (roots)	Cas9	*Agrobacterium*	Desiree	Mutants (84%), *StMYB44* negatively regulates Pi transport by suppressing *StPHO1* gene expression	Zhou et al. (2017)
Tuber quality traits						
*GBBS*	Starch quality	Cas9	Protoplasts (PEG)	Kuras	Multiple allele mutants (67%) and amylopectin-rich and waxy potato	Andersson et al. (2017)
*GBBS*	Starch quality	Cas9/RNP	Protoplasts	Kuras	Regenerants without transgenes (9%)	Andersson et al. (2018)
*GBBS*	Starch quality	Cas9	Protoplasts	Desiree and Wotan	Mutants (35%)	Johansen et al. (2019)
*GBSS I*	Starch quality	Cas9	*Agrobacterium*	Sayaka	Mutants with all four alleles (25%), low amylose starch	Kusano et al. (2018)
*GBBS I*	Starch quality	Cas9	*Agrobacterium*	Desiree	Tetra-allelic mutants by knockout of amylose-producing *StGBSSI* gene	Veillet et al. (2019a)
*Starch synthase gene* (*StSS6*)	Starch biosynthesis	Cas9	*Agrobacterium*	Desiree	Specific gRNA design and successful knock-out *SS6*	Sevestre et al. (2020)
Starch-branching enzymes (SBEs) genes *SBE1*, *SBE2*	Starch quality	Cas9	*Agrobacterium* and protoplasts (PEG)	Desiree	Mutants with valuable starch properties	Tuncel et al. (2019)
*SBE1*, *SBE2*	Starch quality	Cas9/RNP	Protoplasts	Desiree	Three to four allele mutants (72%) with amylase starch with no branching	Zhao et al. (2021)
*PHYTOENE desaturase* (*PDS*)	Carotenoid biosynthesis	Cas9	*Agrobacterium*	Desiree	Mutants (2–10%)	Bánfalvi et al. (2020)
*StPDS*	Carotenoid biosynthesis	Cas9	*Agrobacterium rhizogenes*	Diploid, self-compatible F_1_ hybrid DMF1 (DM1-3 × M6)	Transgenic hairy roots mutants (64–98%)	Butler et al. (2020)
*PDS* and *coilin*	Carotenoid biosynthesis	Cas9	*In vitro* study without delivery	Chicago	Stimulated activity *in vitro*	Khromov et al. (2018)
*St16DOX*	Glycoalkaloids	Cas9	*A. rhizogenes* (electroporation)	May Queen	Full knockout of steroidal glycoalkaloids	Nakayasu et al. (2018)
*Sterol side chain reductase 2* (*StSSR2*)	Steroidal glycoalkaloids (SGAs)	Cas9	*Agrobacterium*	Atlantic	Mutants (64%) with significantly reduced SGAs	Zheng et al. (2021)
Polyphenol oxidases (PPOs) gene (*StPPO2*)	Enzymatic browning	Cas9/RNP	Protoplasts	Desiree	Mutants (69% in four alleles) with 73% reduction in PPO activity than the control	González et al. (2020)
Other traits						
*StDMR6-1* and *StGBSSI*	Phenotype	Cas9	*Agrobacterium*	Desiree	SpCas9-NG application in genome editing	Veillet et al. (2020a)
*StIAA2*	Phenotype	Cas9	*Agrobacterium*	*S. tuberosum* Gp Phureja double monoploid	Mono- and bi-allelic homozygous mutants (83%)	Wang et al. (2015)
*Acetolactate synthase1* (*StALS1*)	Herbicide tolerance	Cas9	*Agrobacterium* and Geminivirus replicon (GVR)	Desiree, diploid (MSX914-10)	Targeted mutants (87–100%)	Butler et al. (2015)
*StALS*	Herbicide tolerance	Cas9	*Agrobacterium* and GVR	Desiree, diploid (MSX914-10)	Improved homozygous recombinants but no change in nonhomologous end joining	Butler et al. (2016)
*StALS1* and *StALS2*	Herbicide tolerance	Cas9/CBE (cytidine base editing)	*Agrobacterium*	Desiree	Transgene-free mutants (10%)	Veillet et al. (2019b)
*StALS1* and *StALS2*	Herbicide tolerance	Cas9/prime editing	*Agrobacterium*	Desiree	Successful prime editing in potato with nucleotide transition/transversion	Veillet et al. (2020b)
*Stylar ribonuclease gene* (*S-RNase*)	Self-incompatibility	Cas9	*Agrobacterium*	DRH-195 and DRH-310 F1	Stable self-compatible mutants through *S-RNase* gene knockout	Enciso-Rodriguez et al. (2019)
*S-RNase*	Self-incompatibility	Cas9	*Agrobacterium*	*S. tuberosum*	Knock out of *S-RNase* gene resulted in self-compatibility	Ye et al. (2018)
				Gp Phureja		
				S15-65		
*NbFT*, *NbPDS3*, and *NbXT2B*	Virus-induced genome editing (VIGE)	Cas9	*Agrobacterium*	Solanaceous plants	Heritable mutants expressing multiple sgRNAs in *Nicotiana benthamiana*/potato	Uranga et al., 2021

*GBBS, Granule-bound starch synthase gene*; PEG, polyethylene glycol; RNP, Ribonucleo protein.

## Application of CRISPR/Cas in Potato

### Biotic and Abiotic Stress Resistance/Tolerance Traits

CRISPR/Cas has emerged as an alternative and efficient technology to accelerate potato breeding (Table 1). It has been demonstrated for potato virus Y (PVY) and late blight (*Phytophthora infestans*) resistance in potato. Cas13a protein was deployed to confer resistance to three PVY strains (RNA virus) by targeting *P3*, *CI*, *Nib*, and *CP* viral genes (Zhan et al., 2019). Host genes like the eukaryotic translation initiation factor *eIF4E* and *coilin* have also been found very effective for PVY resistance (Makhotenko et al., 2019). Recently, late blight resistance was demonstrated in potato by the knockout of susceptibility genes *StDMR6-1* and *StCHL1* (Kieu et al., 2021) and *Caffeoyl-CoA O-methyltransferase* (*StCCoAOMT*) (Hegde et al., 2021).

Abiotic stresses such as heat, drought, salinity, and cold are very important in potato, but with meagre work that is available in potato so far. Zhou et al. (2017) developed mutants (84%) by manipulating potato MYB transcription factor gene *StMYB44*, which negatively regulates phosphate transport in potato by suppressing *StPHO1* gene expression (Table 1). Considerable research work on abiotic stress has been reported in cereals and other crops, but not in potato. Recently, we have proposed the use of CRISPR/Cas to manipulate N metabolism genes for improving nitrogen use efficiency in potato (Tiwari et al., 2020).

### Tuber Quality, Phenotype, and Other Traits

CRISPR/Cas studies have been reported in potato for traits like improved tuber starch quality (Andersson et al., 2017, 2018; Kusano et al., 2018; Johansen et al., 2019; Tuncel et al., 2019; Veillet et al., 2019a; Sevestre et al., 2020; Zhao et al., 2021), carotenoid biosynthesis (Khromov et al., 2018; Bánfalvi et al., 2020; Butler et al., 2020), glycoalkaloids (Nakayasu et al., 2018; Zheng et al., 2021), and enzymatic browning (González et al., 2020) (Table 1). Functional mutants were developed for variations in phenotype (Wang et al., 2015; Veillet et al., 2020a) and herbicide tolerance (Butler et al., 2015, 2016). Self-compatible regenerants were also produced using Cas9 via *Agrobacterium* (Ye et al., 2018; Enciso-Rodriguez et al., 2019) or virus-induced genome editing (VIGE) (Uranga et al., 2021a; 2021b). Researchers have demonstrated the utility of Cas9 base editing and prime editing tools for herbicide tolerance in potato (Veillet et al., 2019b; 2020a; 2020b; 2020c).

## CRISPR/Cas Delivery and Transformation System and Challenges in Tetraploid Potato

Because potato is a highly amenable crop to tissue culture, transformation methods such as *Agrobacterium*, particle bombardment or biolistic, floral-dip, and protoplasts have been applied to it (Sandhya et al., 2020). The most common *Agrobacterium*-mediated transformation and protoplasts that have been successfully deployed in CRISPR/Cas in potato are sgRNA dicot-origin promoters like *Arabidopsis* (*AtUp*)/potato (*StU6p*)/U3p and plant promoters like *CaMV 35S* (Belhaj et al., 2013). However, the *Agrobacterium*-mediated method cannot be used to deliver ribonucleoprotein (RNP) complexes, and elimination of the Cas9 assembly from the plant genome via selfing or backcrossing is more complicated in genetically complex and vegetatively propagated tetraploid potato (Koltun et al., 2018). In potato, each botanical seed called True Potato Seed (TPS), which is a product of the meiosis process, is genetically different from another seed, hence the maintenance of the clonal identity is very crucial.

To address the above issues, the DNA-free delivery system is an ideal approach using somatic cells, i.e. protoplast. Polyethylene glycol (PEG)–mediated protoplast transformation has been found to be an excellent alternative for the efficient delivery of Cas9/gRNA-RNPs in potato (Andersson et al., 2017). DNA-free preassembled Cas9/gRNA-RNPs were directly delivered into the plant cells to induce mutations (Park and Choe, 2019) and were also demonstrated in lipofection-mediated DNA-free delivery (Liu et al., 2020). But with the establishment of suspension culture, protoplast isolation and regeneration into whole plants are the associated problems of the protoplast system (Sandhya et al., 2020).

VIGE is an emerging approach for CRISPR/Cas9 delivery. VIGE involving plant virus–derived vector such as geminivirus replicon has been demonstrated for fast and efficient delivery of sgRNAs in potato (Butler et al., 2015, 2016). This VIGE system bypasses the requirement of transformation and regeneration of plants which is a time-consuming and tedious process. But the large size of a Cas9 assembly challenges the use of the virus vector, as the length of a foreign insert negatively correlates with the stability of the vector.

Recently, base editing and prime editing are the upgraded and more efficient approaches of Cas9. The programmable base editing technology, like the adenine base editor that coverts A.T to G.C without DNA cleavage, has emerged as a boon for crop improvement (Gaudelli et al., 2017). Catalytically inactive Cas9 variant dCas9 or Cas9-nickase is fused with cytosine or adenosine deaminase domain to introduce the desired point mutations (C to T or A to G) in the target region (Mishra et al., 2020). Veillet et al. (2020c) deployed *Staphylococcus aureus*–cytosine base editor (CRISPR-SaCas9 CBE) to edit *StDMR6-1* in potato. Similarly, herbicide tolerance genes *Acetolactate synthase1* and *Acetolactate synthase2* (*StALS2*) were targeted through Cas9 cytidine base editing and Cas9 prime editing technologies, respectively (Veillet et al., 2019b; 2020b). Ariga et al. (2020) used the potato virus X vector to express a base editor consisting of modified Cas9 fused with cytidine deaminase to introduce the targeted nucleotide substitution in *Nicotiana benthamiana.* However, the size of the base editor is larger than Cas9 and this hindered the delivery into cells by the viral vectors.

Overall, high heterozygosity, tetrasomic inheritance, severe inbreeding depression, and vegetative propagation caused difficulties in the successful application of CRISPR/Cas in tetraploid potato. Furthermore, the selection of suitable sgRNA, robust CRISPR/Cas, and efficient transformation protocols and phenotypes without off targets are the main decisive factors in potato. Currently, gene knockout is a preferred mechanism in plants and even all four alleles were mutated through Cas9 in potato *StGBSS* gene (Andersson et al., 2017). PAM limitation (NGG) is one of the drawbacks of SpCas9, and therefore more diversity in CRISPR/Cas toolbox is necessary (Veillet et al., 2020a).

## Conclusions

Desirable plant phenotypes, biotic/abiotic stress resistance/tolerance, and improved tuber quality traits play key roles in potato. The availability of robust CRISPR/Cas arrays, target genes selection, efficient plant transformation protocols, and minimum off-target mutants are the major issues in tetraploid potato. It is a fact that improvement of multigenic traits is difficult than that of the monogenic traits, particularly in potato, due to polyploidy and clonal propagation. Despite this, considerable success has been achieved in potato for some traits and mostly through the gene knockout or insertion/deletion process. Studies have suggested that the use of multiplexing SpCas9 that can handle single or multiple sgRNA/RNPs via targeting conserved sequences combined with protoplast-mediated transformation is an ideal option in potato. Apart from this, awareness among people and policy makers/regulators would be necessary for the success of genome editing research. Collectively, CRISPR-Cas provides an effective next-generation toolbox for fast potato breeding to achieve sustainable crop yield.
