# Supplementary material for: CRISPR/Cas Genome Editing in Potato: Current Status and Future Perspectives
Source: Front Genet. 2022 Feb 2;13:827808. doi: 10.3389/fgene.2022.827808 (PMC8849127; doi:10.3389/fgene.2022.827808)
Supplement: Supplementary file 1 [file Presentation1.PPTX]

## Slide 1
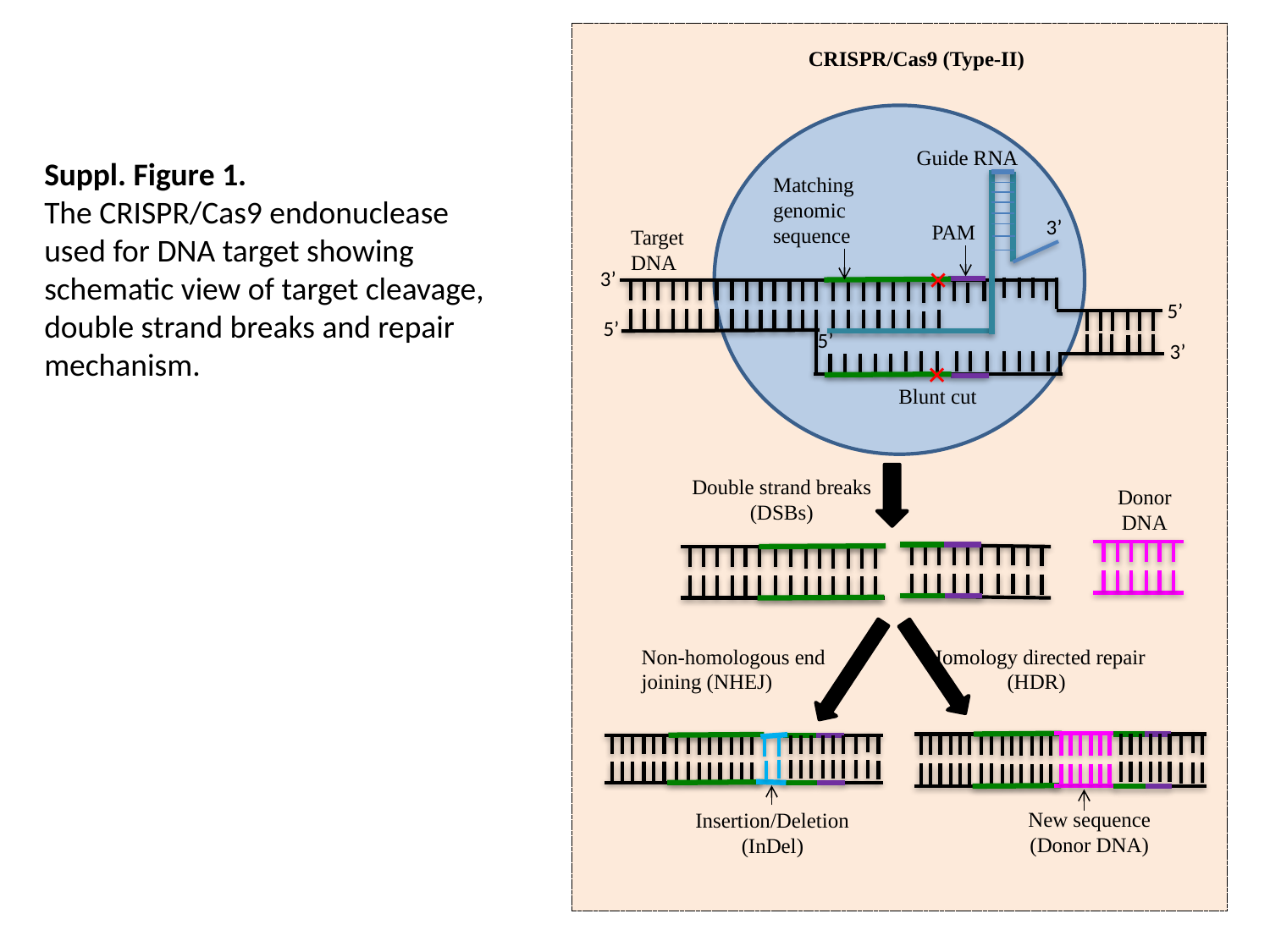

CRISPR/Cas9 (Type-II)
Matching genomic sequence
PAM
Target DNA
Guide RNA
×
×
Blunt cut
Double strand breaks (DSBs)
Donor DNA
Non-homologous end joining (NHEJ)
Homology directed repair (HDR)
Insertion/Deletion (InDel)
New sequence
(Donor DNA)
3’
5’
5’
3’
3’
5’
Suppl. Figure 1.
The CRISPR/Cas9 endonuclease used for DNA target showing schematic view of target cleavage, double strand breaks and repair mechanism.

## Slide 2
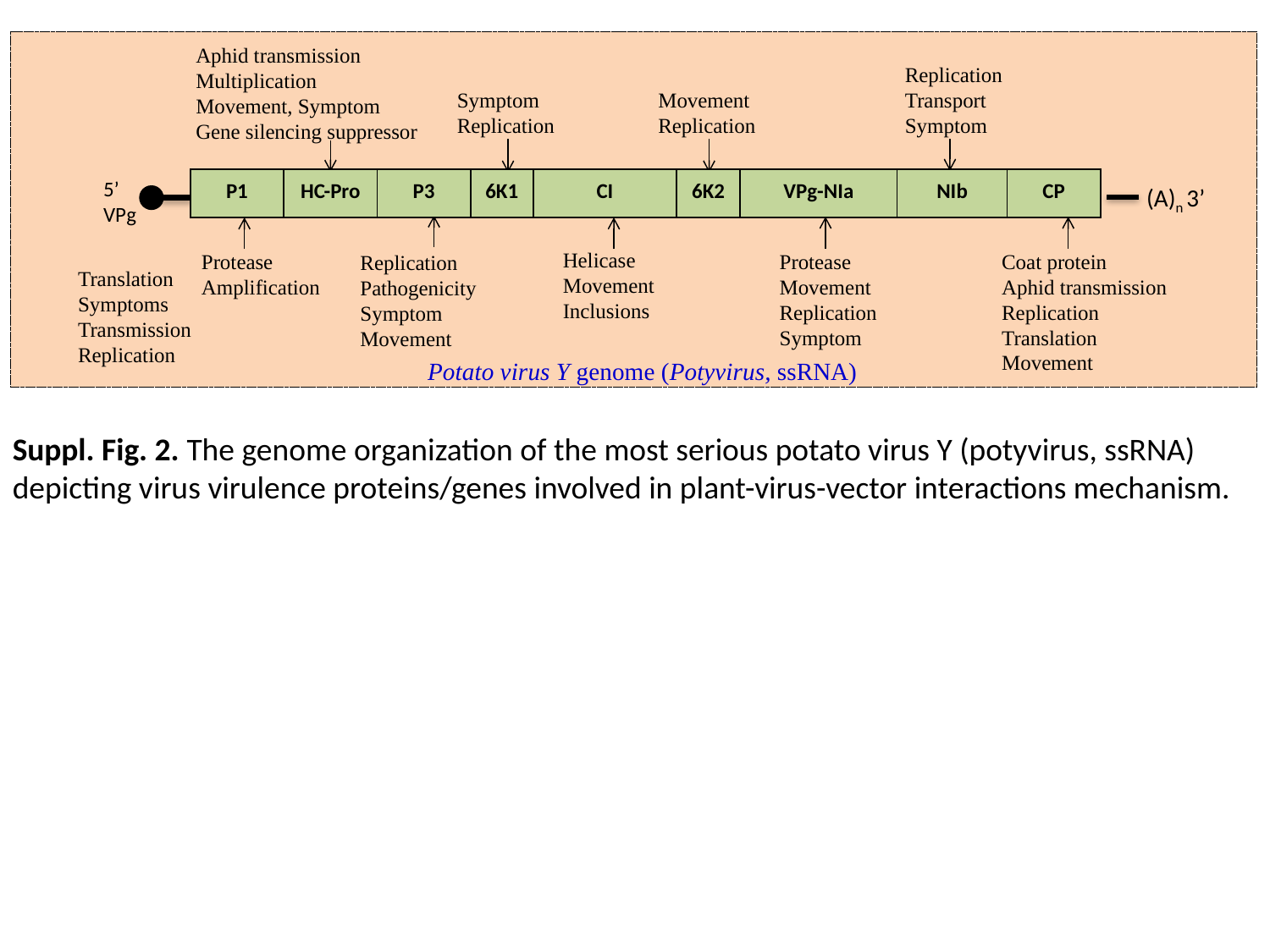

Aphid transmission
Multiplication
Movement, Symptom
Gene silencing suppressor
Replication
Transport
Symptom
Symptom
Replication
Movement
Replication
5’
VPg
| P1 | HC-Pro | P3 | 6K1 | CI | 6K2 | VPg-NIa | NIb | CP |
| --- | --- | --- | --- | --- | --- | --- | --- | --- |
(A)n 3’
Helicase
Movement
Inclusions
Coat protein
Aphid transmission
Replication
Translation
Movement
Protease
Movement
Replication
Symptom
Protease
Amplification
Replication
Pathogenicity
Symptom
Movement
Translation
Symptoms
Transmission
Replication
Potato virus Y genome (Potyvirus, ssRNA)
Suppl. Fig. 2. The genome organization of the most serious potato virus Y (potyvirus, ssRNA) depicting virus virulence proteins/genes involved in plant-virus-vector interactions mechanism.
